# Draft Genome Sequence of a Metronidazole-Resistant *Gardnerella vaginalis* Isolate

**DOI:** 10.1128/genomeA.00992-15

**Published:** 2015-09-03

**Authors:** Jessica A. Schuyler, Sean G. Chadwick, Eli Mordechai, Martin E. Adelson, Scott E. Gygax, David W. Hilbert

**Affiliations:** Femeris Women’s Health Research Center, Medical Diagnostic Laboratories, A Member of Genesis Biotechnology Group, Hamilton, New Jersey, USA

## Abstract

We report the draft genome sequence of a *Gardnerella vaginalis* strain (3549624) isolated from a vaginal specimen. *G. vaginalis* is associated with bacterial vaginosis, the most common cause of vaginal discharge, which is often treated with metronidazole. This isolate is highly resistant to metronidazole (MIC, 500 µg/ml) and may be useful for comparative genomic studies to determine the molecular basis of metronidazole resistance in this species.

## GENOME ANNOUNCEMENT

*Gardnerella vaginalis* was the first organism (1) associated with bacterial vaginosis (BV), the most common gynecological infection in the United States, affecting 29% of women (2). The antibiotic metronidazole is a first-line therapy for BV (3), but treatment failure (4) and recurrent disease are common (5). Metronidazole resistance is observed in clinical *G. vaginalis* isolates (6–8), although it has not been definitively linked to BV treatment failure or recurrent disease. An MIC of ≥32 µg/ml has been used in prior studies to define resistance (8). To facilitate a greater understanding of metronidazole resistance in this species, we sequenced the genome of *G. vaginalis* strain 3549624, an isolate highly resistant to metronidazole (MIC, 500 µg/ml.).

This strain was isolated from a discarded cervicovaginal swab cultured onto human blood Tween (HBT) bilayer medium (Becton Dickson). Gram staining and PCR amplification and sequencing of 16s rDNA were used to confirm the isolate as *Gardnerella vaginalis*. The metronidazole MIC was determined using a metronidazole Etest strip (bioMérieux). Genomic DNA was isolated using a Gentra PureGene kit (Qiagen). Genomic DNA was sequenced using the 454-GS Junior System (Roche) following the manufacturer’s instructions. Genome assembly was performed using the GS *de novo* assembler version 3.0. Annotation was performed using the NCBI Prokaryotic Genome Annotation Pipeline (http://www.ncbi.nlm.nih.gov/genome/annotation_prok).

We generated 111,275 reads totaling 44,322,233 nucleotides. These reads were assembled into a draft genome of 1,732,251 nucleotides at 26-fold coverage. The genome encodes 1,399 genes, 5 rRNA operons, 45 tRNA genes, 67 pseudogenes, 1 noncoding RNA (ncRNA), and 1 clustered regularly interspaced short palindromic repeat (CRISPR) array. *G. vaginalis* strains can be categorized by biotype, based on beta-galactosidase, lipase, and hippurate hydrolase activity (9). Based on the presence of annotated genes encoding enzymes with these three activities (KMT46314.1, KMT46323.1, and KMT46748.1, respectively), we conclude that strain 3549624 is a biotype 1 isolate. *G. vaginalis* has a population structure that consists of four clades (10). The presence of genes encoding alpha-l-fucosidase (KMT46494.1) and galactokinase (KMT46262.1) indicates that this strain belongs to clade 1 (11). Comparison of this genome using GS Reference Mapper version 3.0 to that of ATCC 14019 (GenBank accession number NC_014644.1), another biotype 1 and clade 1 isolate, found 20,333 high-confidence differences (i.e., nucleotide polymorphisms) as well as structural variants, including 48 deletions, 19 duplications, 18 insertions, and 32 inversions, indicating that there is substantial genetic diversity even within the same *G. vaginalis* biotype and clade. Further analysis of this genome in comparison to other sequenced *G. vaginalis* genomes may provide insight into the molecular basis of metronidazole resistance in this species.

### Nucleotide sequence accession numbers.

This whole-genome shotgun project has been deposited in GenBank under the accession number LFWD00000000. The version described in this paper is the first version, LFWD00000000.1.

## References

[1] Gardner HL, Dukes CD 1955 *Haemophilus vaginalis* vaginitis: a newly defined specific infection previously classified non-specific vaginitis. Am J Obstet Gynecol 69:962–976. 14361525

[2] Koumans EH, Sternberg M, Bruce C, McQuillan G, Kendrick J, Sutton M, Markowitz LE 2007 The prevalence of bacterial vaginosis in the United States, 2001–2004: associations with symptoms, sexual behaviors, and reproductive health. Sex Transm Dis 34:864–869. doi:10.1097/OLQ.0b013e318074e565. 17621244

[3] Workowski KA, Berman S 2010 Sexually transmitted diseases treatment guidelines, 2010. MMWR Recomm Rep 59:1–110. 16888612

[4] Koumans EH, Markowitz LE, Hogan V, CDC BV Working Group 2002 Indications for therapy and treatment recommendations for bacterial vaginosis in nonpregnant and pregnant women: a synthesis of data. Clin Infect Dis 35(Suppl 2):S152–S172. 12353202 10.1086/342103

[5] Bradshaw CS, Morton AN, Hocking J, Garland SM, Morris MB, Moss LM, Horvath LB, Kuzevska I, Fairley CK 2006 High recurrence rates of bacterial vaginosis over the course of 12 months after oral metronidazole therapy and factors associated with recurrence. J Infect Dis 193:1478–1486. doi:10.1086/503780. 16652274

[6] Simoes JA, Aroutcheva A, Heimler I, Shott S, Faro S 2001 Bacteriocin susceptibility of *Gardnerella vaginalis* and its relationship to biotype, genotype, and metronidazole susceptibility. Am J Obstet Gynecol 185:1186–1190. doi:10.1067/mob.2001.118144. 11717655

[7] Kharsany AB, Hoosen AA, Van den Ende J 1993 Antimicrobial susceptibilities of *Gardnerella vaginalis*. Antimicrob Agents Chemother 37:2733–2735. doi:10.1128/AAC.37.12.2733. 8109944 PMC192794

[8] Goldstein EJ, Citron DM, Merriam CV, Warren YA, Tyrrell KL, Fernandez HT 2002 *In vitro* activities of garenoxacin (BMS 284756) against 108 clinical isolates of *Gardnerella vaginalis*. Antimicrob Agents Chemother 46:3995–3996. doi:10.1128/AAC.46.12.3995-3996.2002. 12435709 PMC132768

[9] Piot P, Van Dyck E, Peeters M, Hale J, Totten PA, Holmes KK 1984 Biotypes of *Gardnerella vaginalis*. J Clin Microbiol 20:677–679. 6333436 10.1128/jcm.20.4.677-679.1984 PMC271409

[10] Ahmed A, Earl J, Retchless A, Hillier SL, Rabe LK, Cherpes TL, Powell E, Janto B, Eutsey R, Hiller NL, Boissy R, Dahlgren ME, Hall BG, Costerton JW, Post JC, Hu FZ, Ehrlich GD 2012 Comparative genomic analyses of 17 clinical isolates of *Gardnerella vaginalis* provide evidence of multiple genetically isolated clades consistent with subspeciation into genovars. J Bacteriol 194:3922–3937. doi:10.1128/JB.00056-12. 22609915 PMC3416530

[11] Balashov SV, Mordechai E, Adelson ME, Gygax SE 2014 Identification, quantification and subtyping of *Gardnerella vaginalis* in noncultured clinical vaginal samples by quantitative PCR. J Med Microbiol 63:162–175. doi:10.1099/jmm.0.066407-0. 24200640

